# Ferroptosis: a new perspective on the pathogenesis of radiation-induced cataracts

**DOI:** 10.3389/fpubh.2024.1449216

**Published:** 2024-08-16

**Authors:** Yufu Tang, Hongying Liang, Lixia Su, Xiangming Xue, Jingming Zhan

**Affiliations:** Division of Radiology and Environmental Medicine, China Institute for Radiation Protection, Taiyuan, China

**Keywords:** radiation-induced cataracts, ferroptosis, lens epithelial cells, lipid peroxidation, iron homeostasis

## Abstract

Ionizing radiation is a significant risk factor for cataracts, but the pathogenesis of radiation-induced cataracts remains incompletely understood. Ferroptosis, an iron-dependent form of programmed cell death discovered in recent years, has gained increasing attention for its role in various diseases. This article systematically reviews research progress on ionizing radiation, ferroptosis, age-related cataracts, and radiation-induced cataracts. It proposes the “ferroptosis hypothesis” for the pathogenesis of radiation-induced cataracts. Through ionization and oxidative stress effects, ionizing radiation leads to elevated free iron levels and exacerbated lipid peroxidation in lens cells, activating the ferroptosis pathway and resulting in lens opacity. The involvement of ferroptosis in the development of age-related cataracts suggests that it may also be an important pathogenic mechanism of radiation-induced cataracts. Targeting the ferroptosis pathway may be a novel strategy for preventing and treating radiation-induced cataracts. Furthermore, developing new ferroptosis-specific inhibitors with improved targeting and pharmacokinetic properties is also an essential direction for research on preventing and treating radiation-induced cataracts. The study of ferroptosis provides new insights into the mechanism and management of radiation-induced cataracts, potentially transforming radiation-induced cataracts from “inevitable” to “preventable and treatable.”

## 1 Introduction

Cataract is the leading cause of blindness worldwide, and its incidence continues to rise with the aging population (1). Ionizing radiation (IR) is one of the critical environmental factors contributing to cataract development. IR can be classified into low linear energy transfer (LET) radiation, such as X-rays and gamma rays, and high LET radiation, such as heavy ions and neutrons. Low and high LET radiation can induce cataract development, but their biological effects and risk differ (2). Epidemiological studies have shown that populations exposed to IR, such as radiation workers, nuclear accident survivors, patients receiving head and neck tumor radiotherapy, and astronauts, have a significantly higher incidence of cataracts compared to the general population (3–7).

The biological effects and cataract risks differ between low and high LET radiation. Azizova et al. (4, 8) found that cataract risk significantly increased with higher radiation doses in Mayak workers, with posterior subcapsular cataracts (PSC) exhibiting the highest radiosensitivity. Little et al.’s (6) study on US radiologic technologists revealed that cataract risk was significantly increased even at low radiation doses. Similarly, the French study “Occupational Cataracts and Lens Opacities in Interventional Cardiology” found a substantial increase in PSC risk among interventional cardiologists (7). Neriishi et al. conducted a 19-year follow-up of Japanese atomic bomb survivors, demonstrating a positive correlation between cataract surgery incidence and radiation dose (9). Studies on astronauts have shed light on the profound long-term effects of exposure to the unique space radiation environment characterized by high LET radiation. These investigations have revealed a significantly heightened risk of cataract development among astronauts, particularly those participating in long-duration space missions (10–12). A study on 295 astronauts found that cataracts were significantly higher in astronauts on long-duration space missions than those without. The risk of cataracts was positively correlated with space flight time and space radiation dose (13). Animal experiments have confirmed that high LET radiation, such as heavy ions, can induce more severe and earlier-onset cataracts than low LET radiation (14–16). Factors such as dose rate and fractionation of IR exposure also influence the risk of cataract development (17). Therefore, a deeper understanding of the pathogenic mechanisms of radiation-induced cataracts is crucial for developing effective prevention and treatment strategies.

Radiation-induced cataracts are closely related to the abnormal death of lens epithelial cells (LECs). Studies have shown that through direct ionization or indirect effects mediated by reactive oxygen species (ROS), IR causes DNA damage, protein modification, and lipid peroxidation in LECs, leading to cell apoptosis or necrosis and lens opacity (18). In 2023, Chen et al. found that IR significantly upregulated the mRNA and protein expression of Bax and caspase-3 in rat lenses, accompanied by a decrease in Bcl-2, potentially activating the caspase cascade, initiating the apoptotic program, and playing a pivotal role in ionizing radiation-induced cataracts (19). Lledó et al. discovered that the NLRP3 inflammasome is crucial in ionizing radiation-induced pyroptosis of human LECs (20). However, these mechanisms are insufficient to fully explain the complex pathological process of ionizing radiation-induced cataracts, and further exploration of novel cell death modalities and their regulatory mechanisms is urgently needed.

In recent years, ferroptosis, as a new form of cell death, has gained widespread attention for its role in various diseases such as neurodegenerative disorders, ischemia–reperfusion injury, and cancer (21–23). In both animal models of Parkinson’s disease and brain tissues of patients, elevated expression of ferroptosis markers such as ferritin heavy chain 1 (FTH1) has been found, suggesting that ferroptosis plays an essential role in the pathogenesis of Parkinson’s disease (24). Further research has shown that the iron chelator deferiprone can alleviate cellular ferroptosis and slow the progression of Parkinson’s disease (25–27). In patients with ischemic stroke, ferroptosis inhibitors can reduce brain tissue ferroptosis and infarct size (28). In cancer research, there is growing evidence that inducing tumor cell ferroptosis is an essential mechanism of action for some anticancer drugs such as sorafenib and erastin (23).

Although the crucial role of ferroptosis in various diseases has been widely recognized, its role in ionizing radiation-induced lens damage remains unclear. Given that ferroptosis is closely related to oxidative stress and lipid peroxidation. Oxidative damage caused by IR is an essential mechanism for cataract development. However, the etiologies of radiation-induced cataracts and age-related cataracts differ, they share similarities in histopathological changes in the lens, such as increased epithelial cell apoptosis, liquefaction of lens fiber cells, and aggravated oxidative stress responses (29, 30). This suggests that radiation-induced and age-related cataracts may have common cellular and molecular mechanisms (31, 32). Ferroptosis has been proven to be involved in age-related cataracts, and it is speculated that it may also play a significant role in the development of radiation-induced cataracts.

In summary, as a new development in cell death, ferroptosis provides a new research direction for understanding the pathogenesis of radiation-induced cataracts. An in-depth exploration of the relationship between IR, ferroptosis, and cataract development is expected to reveal new mechanisms of radiation-induced cataracts and provide new strategies for their prevention and control. This article aims to systematically review the latest advances in ferroptosis research and, combined with the characteristics of radiation-induced cataracts, focus on the following scientific questions: (1) the relationship between ionizing radiation-related tissue damage and cellular ferroptosis; (2) the role and mechanism of ferroptosis in age-related cataracts; (3) the possible mechanism of ferroptosis in the development of radiation-induced cataracts; (4) new ideas provided by ferroptosis research for the prevention and treatment of radiation-induced cataracts. To our knowledge, this is the first systematic exploration of the role and mechanism of ferroptosis in radiation-induced cataracts, which provides a new perspective for elucidating the pathogenesis of this disease.

## 2 The relationship between ionizing radiation-related tissue damage and cellular ferroptosis

The relationship between ionizing radiation-related tissue damage and cellular ferroptosis has garnered increasing attention. As a high-energy radiation, IR can cause damage to multiple systems and organs in the body, such as the hematopoietic, digestive, and skin. These injuries not only affect patients’ quality of life but may also induce severe complications. Therefore, understanding the molecular mechanisms underlying ionizing radiation-induced tissue damage is crucial for developing adequate protection and treatment strategies.

Recent studies have revealed the critical role of ferroptosis in ionizing radiation-related tissue damage. Ferroptosis is a newly discovered form of programmed cell death characterized by non-heme iron-dependent lipid peroxidation and elevated ROS levels, leading to cell death. Zhou et al., while investigating ionizing radiation-induced intestinal injury, found that IR can activate Transferrin Receptor 1 (TfR1), promoting iron uptake in intestinal epithelial cells and resulting in abnormally elevated intracellular free iron levels. Excessive free iron generates high levels of ROS through the Fenton reaction, exacerbating lipid peroxidation and inducing ferroptosis in intestinal epithelial cells, ultimately leading to impaired intestinal barrier function. Building on this foundation, researchers further explored the protective effects of ferroptosis inhibition strategies against ionizing radiation-induced intestinal injury. Results showed that the iron chelator deferoxamine (DFO) could effectively chelate excess intracellular free iron, reduce lipid peroxidation levels, inhibit ionizing radiation-induced ferroptosis in intestinal epithelial cells, and thus alleviate radiation-induced intestinal injury (33). This study confirmed the pathological role of ferroptosis in radiation-induced intestinal injury and provided experimental evidence for the application of ferroptosis inhibitors in radiation damage protection.

Ferroptosis-mediated radiation damage is not limited to the intestine; it also plays a vital role in tissues such as the skin and brain. Wang et al. systematically investigated the relationship between IR, ferroptosis, and skin damage using a rat model of radiation-induced skin injury. Results showed that IR significantly increased ferroptosis levels in skin tissue by upregulating the expression of crucial ferroptosis genes such as acyl-CoA synthetase long-chain family member 4 (ACSL4), leading to radiation-induced skin damage manifestations such as epidermal atrophy and dermal fibrosis. Inhibition of ferroptosis reduced the severity of radiation-induced skin tissue damage (34). In a mouse model of radiation-induced brain injury, researchers detected significantly elevated levels of neuronal ferroptosis. Mechanistic studies revealed that IR upregulated the expression of ferroptosis effector molecules such as ferritin heavy chain (FTH), ferritin light chain (FTL), and TfR1 while downregulating glutathione peroxidase 4 (GPX4) expression, inducing ferroptosis in neuronal cells and causing blood–brain barrier disruption (35). These studies further support the notion that ferroptosis may be a common mechanism underlying ionizing radiation-induced multi-organ damage.

In summary, ferroptosis plays a crucial role in ionizing radiation-related tissue damage. IR induces ferroptosis in various tissues by disrupting intracellular iron homeostasis, lipid metabolism, and oxidative stress, leading to pathological organ damage. These findings provide a solid foundation for exploring the potential mechanisms of ferroptosis in the pathogenesis of radiation-induced cataracts, as the LECs, the main target of IR in the lens, are likely to undergo ferroptosis in response to radiation-induced oxidative stress, lipid peroxidation, and iron dysregulation. In the following sections, we will delve into the role and mechanisms of ferroptosis in age-related cataracts and discuss the potential implications for understanding radiation-induced cataracts.

## 3 The role and mechanisms of ferroptosis in age-related cataracts

Age-related cataracts, the leading cause of blindness in the older adults, exhibit a significantly increased prevalence with advancing age. Currently, surgical replacement of the opacified lens remains the primary treatment for cataracts; however, the risk of postoperative complications is high, severely impacting patients’ quality of life. Therefore, elucidating the pathogenic mechanisms of age-related cataracts and exploring novel preventive and therapeutic strategies are crucial for reducing the burden of this disease.

Traditionally, the development of age-related cataracts has been attributed to factors such as endogenous oxidative stress, protein denaturation and aggregation, and impaired nutrient transport within the lens (36). However, these theories fail to explain the pathological process of cataracts fully, and many molecular mechanisms remain to be further elucidated. In recent years, growing evidence suggests that ferroptosis plays a significant role in the onset and progression of age-related cataracts.

Previous studies have demonstrated that ferroptosis has important pathological implications in various age-related diseases, including neurodegenerative disorders and cardiovascular diseases (21, 37). This raises the question of whether ferroptosis is also involved in developing age-related cataracts.

To address this question, Wei et al. analyzed key markers of ferroptosis in aged human LECs. The results revealed significantly decreased expression levels of cystine/glutamate antiporter (SLC7A11), solute carrier family three member 2 (SLC3A2), and ferroportin (SLC40A1) in aged LECs (38), suggesting that age-related cataracts are highly sensitive to ferroptosis.

Ma et al. established a mouse aging model to validate this hypothesis further and confirmed that ferroptosis occurs in aged mouse LECs. Subsequent *in vitro* experiments were conducted, and ferroptosis inhibitor Ferrostatin-1 (Fer-1) treatment significantly enhanced cell viability (39). This study provided experimental evidence supporting the involvement of ferroptosis in the progression of age-related cataracts and the potential application of ferroptosis inhibition strategies in cataract prevention and treatment.

Building upon the evidence that ferroptosis contributes to the development of age-related cataracts, researchers further explored the underlying molecular mechanisms. Mechanistic studies revealed that the expression of iron regulatory protein 2 (IRP2), a key regulator of iron homeostasis, is downregulated in age-related cataracts, leading to abnormally elevated free iron levels in the lens (40). Excessive free iron generates many highly reactive hydroxyl radicals through the Fenton reaction, exacerbating lipid peroxidation. Simultaneously, arachidonate 15-lipoxygenase (ALOX15) catalyzes the peroxidation of unsaturated fatty acids, further aggravating lipid peroxidation damage (41), which may ultimately activate downstream ferroptosis pathways, causing LECs death and lens opacification.

Furthermore, research has found that the aging process in the lens is accompanied by a decline in autophagic function (42). Autophagy is a crucial cell pathway to remove damaged proteins and senescent organelles. Impaired autophagic function leads to the accumulation of ferroptosis substrates, such as lipid peroxides and reactive iron, within the lens, further promoting the occurrence of ferroptosis (43). This suggests that restoring autophagic function in lens cells may be another potential strategy for inhibiting ferroptosis and preventing age-related cataracts.

The findings from studies on ferroptosis in age-related cataracts provide valuable insights into the potential role of ferroptosis in radiation-induced cataracts, as both types share common pathogenic factors such as oxidative stress and lipid peroxidation. The mechanisms of ferroptosis discovered in age-related cataracts, including iron dysregulation, ROS accumulation, and impaired antioxidant defenses, are likely to be involved in the development of radiation-induced cataracts. In the next section, we will explore the potential mechanisms of ferroptosis in radiation-induced cataracts, building upon the knowledge gained from studies on ferroptosis in age-related cataracts and ionizing radiation-related tissue damage.

## 4 Potential mechanisms of ferroptosis in the development of radiation-induced cataracts

Radiation-induced cataracts are a lens-damaging disease caused by IR, commonly observed in tumor patients receiving head and neck radiotherapy and high-radiation occupational populations such as nuclear plant workers. The pathogenesis of radiation-induced cataracts is not fully elucidated, and effective prevention and treatment methods are lacking. Given that IR can induce ferroptosis in various tissue cells. Ferroptosis is involved in age-related cataract pathology, and it is hypothesized that ferroptosis may also be an essential mechanism underlying radiation-induced cataract development.

**Figure 1:**
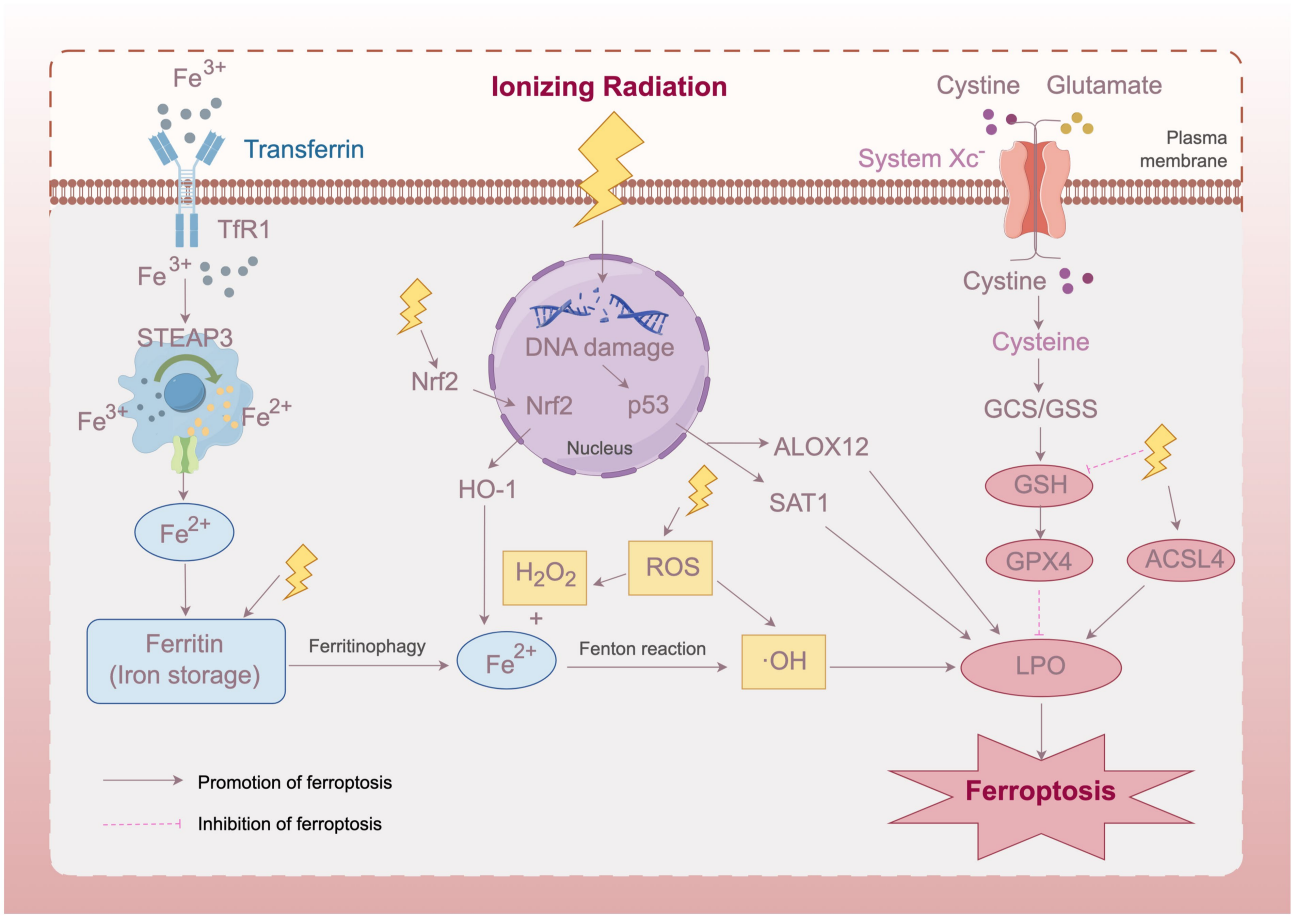
Schematic diagram of the mechanism of ferroptosis in radiation-induced cataracts. IR induces ferroptosis in lens epithelial cells through multiple pathways, including elevated free iron levels, activated ferroptosis-related transcription factors, and inhibited GPX4 activity, ultimately leading to lens opacification. Transferrin Receptor 1 (TfR1), Six-Transmembrane Epithelial Antigen of Prostate 3 (STEAP3), Nuclear factor erythroid 2-related factor 2 (Nrf2), Heme Oxygenase-1 (HO-1), Reactive Oxygen Species (ROS), Arachidonate 12-Lipoxygenase, 12S Type (ALOX12), Spermidine/Spermine N1-Acetyltransferase 1 (SAT1), Gamma-Glutamylcysteine Synthetase/Glutathione Synthetase (GCS/GSS), Glutathione (GSH), Glutathione Peroxidase 4 (GPX4), Acyl-CoA Synthetase Long Chain Family Member 4 (ACSL4), Lipid Peroxidation (LPO). This figure was created using Figdraw.

Based on current research findings, we propose a schematic diagram of the mechanism of ferroptosis in radiation-induced cataracts (Figure 1). IR elevates free iron levels in lens cells by directly degrading ferritin and activating heme oxygenase-1 (HO-1) to break down heme, releasing ferrous iron (Fe^2+^). Excessive free iron generates hydroxyl radicals through the Fenton reaction, aggravating lipid peroxidation. Meanwhile, radiation activates ferroptosis-related transcription factors such as p53, upregulating the expression of ferroptosis-effector genes and promoting lipid peroxidation. Additionally, IR depletes glutathione (GSH) and consequently inhibits the expression and activity of GPX4, weakening the cell’s ability to resist lipid peroxidation. Multiple mechanisms jointly activate the ferroptosis pathway, leading to LECs death and opacification.

### 4.1 Ionizing radiation leads to abnormally elevated free iron levels in lens cells

As shown in Figure 1, IR could potentially lead to abnormally elevated free iron levels in lens cells through direct and indirect ionization effects. On the one hand, IR can cause ferritin to release stored iron, increasing free iron content (44); on the other hand, HO-1 induced by IR can catabolize heme, resulting in increased free iron release (30, 45). Excessive free iron generates high levels of ROS through the Fenton reaction, exacerbating lipid peroxidation and ultimately inducing ferroptosis (46).

The degree of free iron elevation and the severity of subsequent ferroptosis may vary depending on the type and dose of IR. High LET radiation, such as heavy ions and neutrons, deposits more energy along its track, generating denser ionization events than low LET radiation like X-rays and gamma rays. Consequently, high LET radiation may cause more extensive damage to ferritin and iron-containing proteins, leading to a more significant increase in free iron levels. This notion is supported by studies showing that high LET radiation induces more severe oxidative damage and cell death than low LET radiation at the same dose (14, 47).

### 4.2 Effects of different ROS types on lens cell ferroptosis

The types of ROS generated by IR also play a crucial role in determining the extent of ferroptosis in lens cells. High LET radiation generates more reactive ROS, such as hydroxyl radical (•OH), while low LET radiation tends to produce less reactive ROS, such as superoxide anion (O_2_^•−^) and hydrogen peroxide (H_2_O_2_) (48, 49). Due to their more substantial oxidizing properties, highly reactive ROS can cause more severe oxidative damage to biological macromolecules such as proteins, lipids, and DNA, accelerating lens cell death and cataract development (50).

From the perspective of the ferroptosis theory, different types of ROS may have different effects on the initiation and execution of ferroptosis in lens cells. Studies have shown that •OH can directly trigger ferroptosis by inducing lipid peroxidation (51). H_2_O_2_ can oxidize Fe^2+^ to produce •OH through the Fenton reaction, thereby promoting ferroptosis (52). Therefore, the types of ROS generated by different radiation may influence the occurrence and extent of ferroptosis in lens cells. Low LET radiation may induce a progressive ferroptosis by accumulating less reactive ROS. In contrast, high LET radiation may trigger rapid and severe ferroptosis due to highly reactive ROS. The specific ROS thresholds for inducing ferroptosis in lens cells and the in-depth molecular mechanisms need further investigation in future studies.

### 4.3 Ionizing radiation induces ferroptosis through transcriptional regulation and suppression of antioxidant defense

In addition to directly modulating free iron levels and ROS production, IR can induce ferroptosis through transcriptional regulation of ferroptosis-related genes. Radiation exposure activates transcription factors such as p53 (53), which regulate the expression of pro-ferroptotic genes involved in iron metabolism and lipid peroxidation. P53 induces lipoxygenase (ALOX12) and spermine/spermidine N1-acetyltransferase (SAT1), promoting lipid peroxidation (54, 55).

Moreover, IR impairs the cell’s antioxidant defense system, particularly GPX4, further exacerbating ferroptosis. Radiation upregulates GPX4’s endogenous inhibitor, ACSL4, leading to GPX4 inactivation and subsequent lipid peroxide accumulation. Simultaneously, IR depletes GSH, the essential cofactor for GPX4, further compromising its ferroptosis-suppressing function (19, 56, 57).

The relative contributions of iron dysregulation, ROS production, transcriptional regulation, and antioxidant defense impairment to ferroptosis in irradiated lens cells may depend on factors such as radiation type, dose, and duration of exposure. High LET radiation’s dense ionization may cause more immediate iron release and ROS generation, while low LET radiation may rely more on the gradual transcriptional changes and GSH depletion to induce ferroptosis over time. These mechanisms likely work in synergy to drive ferroptosis in irradiated lens cells, with their relative weights varying depending on the specific radiation exposure conditions.

Unraveling the complex interplay between these mechanisms and their dose–response relationships will be crucial for developing mechanism-based interventions to prevent and treat radiation-induced cataracts. Future studies should focus on quantitatively assessing the contributions of each mechanism to ferroptosis in lens cells under different radiation exposure scenarios and identifying the key molecular targets for intervention. This will lay the foundation for developing targeted ferroptosis-modulating strategies to mitigate radiation-induced cataract risk and progression.

## 5 New insights provided by ferroptosis research for the prevention and treatment of radiation-induced cataracts

Ferroptosis research provides new ideas and strategies for preventing and treating radiation-induced cataracts. Traditionally, the primary means of preventing ionizing radiation-induced cataracts has been to minimize unnecessary radiation exposure (58), while effective therapeutic drugs for established radiation-induced cataracts have been lacking (59, 60). As our understanding of the role of ferroptosis in the pathogenesis of radiation-induced cataracts deepens, targeted regulation of ferroptosis pathways is becoming a promising new approach for preventing and controlling radiation-induced cataracts.

Animal studies have demonstrated that DFO attenuates radiation-induced small intestine and skin injuries (61, 62), suggesting that iron chelators may have a radioprotective effect. Therefore, it is necessary to evaluate the preventive efficacy of ferroptosis chelators/inhibitors against radiation-induced cataracts in radiation workers or tumor patients receiving head and neck radiotherapy.

Treatment with ferroptosis inhibitors or iron chelators is also a potential strategy for established radiation-induced cataracts. These drugs may slow cataract progression by blocking persistently activated ferroptosis pathways in lens cells, inhibiting iron-dependent lipid peroxidation, and maintaining intracellular redox homeostasis. An *in vitro* study found that Fer-1 inhibits NaIO_3_-induced ferroptosis in human LECs, maintaining cell survival (39). Considering the persistent oxidative stress and ferroptosis in the lenses of patients with radiation-induced cataracts, late-stage therapeutic administration of ferroptosis inhibitors or iron chelators may be beneficial. Future research should focus on pharmacodynamic evaluations to determine the optimal timing, dosage, and duration of drug administration to maximize therapeutic efficacy.

Currently, known iron chelators such as deferiprone and deferoxamine primarily chelate-free iron, and their effects on ferroptosis pathways are still limited. Therefore, developing novel ferroptosis inhibitors with enhanced targeting and specificity is an essential direction for research on preventing and treating radiation-induced cataracts. For example, small molecule drugs that specifically inhibit ferroptosis effector proteins such as ACSL4 could be designed, or medications that specifically activate Nrf2 or inhibit p53, which are transcriptional regulators of ferroptosis, could be developed to modulate ferroptosis pathways at multiple levels more precisely. Additionally, drugs could be combined with lens-specific delivery systems to increase drug concentration and retention time in lens tissue while reducing systemic side effects (63). These drug development strategies will propel the prevention and treatment of radiation-induced cataracts from “vague” to “precise.”

## 6 Conclusion

This review proposes the “ferroptosis theory,” elucidating a novel mechanism by which IR induces radiation-induced cataracts through ferroptosis in lens cells. Targeting ferroptosis pathways may become a new strategy for preventing and treating radiation-induced cataracts. However, further research is needed to fully understand the ferroptosis mechanisms in radiation-induced cataracts, such as the specific mechanisms of radiation-induced iron homeostasis disruption, the interaction between ferroptosis and other cell death modalities, and the establishment of ferroptosis biomarkers and detection methods.

Future studies should leverage various techniques, including omics, imaging, and animal models, to investigate the molecular mechanisms of ionizing radiation-induced ferroptosis in the lens and clarify the role of ferroptosis at different stages of radiation-induced cataract development. Simultaneously, efforts should be made to develop and optimize specific ferroptosis inhibitors, enhancing their targeting ability and pharmacokinetic properties to provide new drug options for the precise prevention and control of radiation-induced cataracts. Moreover, large-scale epidemiological surveys and cohort studies should be conducted to evaluate the predictive and diagnostic value of ferroptosis-related biomarkers for radiation-induced cataracts, providing a basis for early screening and intervention in high-risk populations.

In conclusion, ferroptosis research opens new directions and paths for studying the pathogenesis and prevention strategies of radiation-induced cataracts. It ultimately aims to shift radiation-induced cataracts from “inevitable” to “preventable and treatable,” safeguarding the visual health of radiation-related populations. The study of ferroptosis in radiation-induced cataracts is a long and arduous task, requiring collaborative innovation and tackling critical problems across multiple disciplines, including ophthalmology, radiation medicine, and pharmacology, to open up new frontiers in the prevention and treatment of radiation-induced cataracts.
